# Changes in Health Education Literacy After Structured Web-Based Education Versus Self-Directed Online Information Seeking in Patients Undergoing Carpal Tunnel Release Surgery: Nonrandomized, Controlled Study

**DOI:** 10.2196/65114

**Published:** 2025-03-25

**Authors:** Mariella Seel, Julian Alexander Mihalic, Stefan Mathias Froschauer, Bernhard Holzner, Jens Meier, Tobias Gotterbarm, Matthias Holzbauer

**Affiliations:** 1 Faculty of Medicine, Johannes Kepler University of Linz, Linz, Austria; 2 Department for Orthopedics and Traumatology, Kepler University Hospital GmbH, Krankenhausstrasse 9, Linz, 4020, Austria; 3 Department of Psychiatry, Psychotherapy, Psychosomatics and Medical Psychology, University Hospital of Psychiatry II, Innsbruck Medical University, Innsbruck, Austria; 4 Department of Anesthesiology and Intensive Care Medicine, Kepler University Hospital GmbH, Johannes Kepler University of Linz, Linz, Austria

**Keywords:** carpal tunnel release surgery, patient education, structured web-based education, online information, health education literacy, web-based, health education, information seeking, carpal tunnel release, carpal tunnel surgery, non-randomized, controlled study, self-management, perioperative, online health information, health literacy, day surgery, online search, carpal tunnel, carpal

## Abstract

**Background:**

With advancements in anesthesiologic and surgical techniques, many surgeries are now performed as day-surgery procedures, requiring greater responsibilities for self-management from patients during the perioperative process. Online health information often lacks reliability and comprehensibility, posing risks for patients with low health literacy. Carpal tunnel release (CTR) surgery, a common day-surgery procedure, necessitates effective patient education for optimal recovery and self-management.

**Objective:**

This study introduces the CTS Academy, a web-based education program designed for patients undergoing CTR day surgery. The study aimed to evaluate the CTS Academy’s impact on patients’ health education literacy (HEL) compared with self-directed online information seeking.

**Methods:**

A scoping review on education programs focusing on the perioperative process of CTR was conducted before this study. In a nonrandomized controlled study, 60 patients scheduled for CTR were assigned to 2 groups based on the patients’ preferences; the test group used the CTS Academy, while the control group performed self-directed online searches. HEL was assessed using the Health Education Literacy of Patients with chronic musculoskeletal diseases (HELP) questionnaire, focusing on patients’s comprehension of medical information (COMPR), patients’s ability to apply health-related information in an everyday life (APPLY), and patient’s ability to communicate with health care professional (COMM). Secondary outcomes included content comprehensibility, patient preferences, platform usability, and clinical carpal tunnel syndrome (CTS)–related parameters.

**Results:**

In the scoping review, 17 studies could be identified and included for full-text analysis. Eighteen patients each were included in the test group (13 women and 5 men) and in the control group (11 women and 7 men). The average time spent in the study was 167 and 176 days for the test and control groups, respectively. The test group showed significant improvements in APPLY (mean 28, SD 7.99 vs mean 24, SD 5.14; *P*<.05) and COMM (mean 30, SD 10.52 vs mean 25, SD 6.01; *P=.*02) after using the CTS Academy in a longitudinal analysis. No significant changes were observed in the control group. In a comparison between groups, the test group had significantly higher APPLY scores at follow-up (mean 24, SD 5.14 vs mean 33, SD 14.78; *P=.*044) and fewer comprehension issues at baseline (mean 38, SD 16.60 vs mean 50, SD 19.00; *P=.*03). The CTS-related knowledge assessment yielded 92% (66/72) versus 90% (65/72) correct answers in the test and control groups, respectively. The test group rated the CTS Academy highly in usability (6.22 of 7.00 points) and utility (6.13 of 7.00 points). Preferences leaned toward using CTS Academy alongside doctor consultations (16/18, 89%) and over self-directed searches (15/18, 84%). No significant differences were found in CTS-related symptoms between groups.

**Conclusions:**

The CTS Academy effectively enhanced patients’ HEL, especially in applying and communicating medical information. The platform’s usability and utility were rated favorably, and patients preferred it over independent online information seeking. This suggests that structured, web-based education enhances patient self-management during the day surgery process.

## Introduction

### Background

Due to continuous advancements in surgical and anesthesiologic techniques, an increasing number of surgical interventions are being performed in a day surgery setting. Consequently, patients spend less time in the hospital and are often overwhelmed by a large amount of information regarding their condition and the perioperative process provided during face-to-face interactions with medical professionals [1]. Thus, patients are required to assume greater responsibilities for self-management and self-care at home [1]. However, many patients lack the necessary knowledge and ability to effectively manage the day surgery situation, leading to an increased demand for comprehensive patient education [2]. Patients seeking online health information frequently encounter the risk of unreliable sources and incorrect information due to insufficient quality control or issues with comprehensibility [3]. Considering the low health literacy levels among some patients, this poses a substantial threat to effective health communication and patient safety [4].

Despite the low-risk profile of many ambulatory interventions, the recovery phase poses challenges, emphasizing the need for patient self-management and comprehensive education programs addressing both physical and psychological aspects [1,5] to empower patients. Patient empowerment can be defined as “a process through which people gain greater control over decisions and actions affecting their health” [6]. It can be achieved through patient education, a “process of assisting consumers of health care to learn how to incorporate health related behaviors (knowledge, skill, attitude) into everyday life with the purpose of achieving the goal of optimal health” [7]. This approach should provide patients with the health-related knowledge and skills to manage their medical conditions effectively [8]. Closely related is the concept of health education literacy (HEL), a patient’s “cognitive and social skills […] to understand and use the information as conveyed in health education programmes” [9]. HEL is based on the concept of health literacy and consists of three dimensions, that are (1) the patient’s ability to apply health-related information in an everyday life (APPLY), (2) the patient’s ability to communicate with health care professionals (COMM), and (3) the patient’s comprehension of medical information (COMPR) [9].

### Goal of This Study

Open carpal tunnel release (CTR) surgery is a common day-surgery procedure for carpal tunnel syndrome (CTS) [10]. CTS affects approximately 3% of the general adult population [11] and is caused by compression of the median nerve and results in symptoms such as weakness, tingling, and pain [12]. In a recent meta-analysis, CTR as gold standard treatment results in reliable improvements in hand function and symptom severity on the long-term follow-up, while manual therapy only provides short term effects regarding pain relief [13]. To address time constraints and information overload experienced by patients during clinical visits and to enhance their comprehension of their medical condition and treatment, we aim to introduce the CTS Academy, a patient-centered web-based education program, in this study. Since the implementation of patient-centered web-based education programs focusing on the perioperative process of CTR is rare, a scoping review was conducted before this study to highlight recent gaps in existing research. Our program is tailored to the specific needs of patients undergoing day surgery for CTR and aims to provide valid and comprehensible information in a familiar environment, thereby facilitating patient self-management.

In a nonrandomized controlled study, we aimed to evaluate the CTS Academy for its effect on patients’ HEL compared with self-directed online information seeking, 3 weeks after CTR (in comparison with the baseline survey).

We hypothesize that HEL is increased by participating in a web-based, structured educational programme (CTS Academy) for patients undergoing CTR*.* Furthermore, we examine the comprehensibility of educational content, patients’ preferences in education, the usability of the CTS Academy platform, and clinical changes in CTS-related parameters. Our research addresses the scarcity of comparable implementations and aims to acquire further insights into the existing limited quantitative research in this domain.

## Methods

### Scoping Review of the Literature

Before the conduction of this study, we performed a scoping review of literature discussing the quality of online CTS information. From September to December 2022, we searched Epistemonikos, the Cochrane Library, PubMed, Connected Papers, and Google Scholar. Search prompts were for example “perioperative education,” “web-based,” “carpal tunnel release,” and “structured patient education,” and the complete search strategy can be requested from the authors. The search aimed to identify systematic reviews, original research, and related studies on web-based patient education and online information for patients undergoing open CTR surgery. No filters were applied for publication date or status, but only English and German full texts were analyzed. Publications with low evidence levels or lacking full study descriptions were excluded, while a broad search strategy ensured relevant materials not explicitly titled as web-based education were included. Reporting was performed based on the Joanna Briggs Institute Scoping Review Reporting Template [14] and the PRISMA-ScR (Preferred Reporting Items for Systematic reviews and Meta-Analyses extension for Scoping Reviews) Checklist [15].

### Design of the CTS Academy

We implemented the CTS Academy platform prototype using Microsoft Sway [16], a freely accessible web-based software for creating single-page websites.

The target group for this program consists of patients scheduled for open CTR day surgery who seek relevant information related to physical and mental preparation for CTR surgery.

The goal of CTS Academy is to assist patients preparing for surgery and managing the postoperative phase by providing textual information, multimedia content, self-check quizzes, downloadable or printable checklists for patients’ self-directed online learning.

In the CTS Academy, we primarily focus on biophysiological, functional, experiential, and social aspects, excluding financial information due to the Austrian public health insurance system. The ethical and cognitive perspectives, emphasizing patient recognition, appreciation, and knowledge form the foundation of the CTS Academy. Patient education programs that prioritize patient-centeredness and engagement rely on evidence-based strategies, including plain language and multimodal information delivery [17]. Keulers et al [18] propose practical implications for interactive patient education, advocating for varied information presentation through text, graphics, and videos, with structured text using headings and subheadings. Summaries of lengthy texts determine the relevance of content, and the inclusion of graphs, pictures, and patient role models, such as animated characters, aids comprehension [18]. Incorporating entertaining elements, such as storytelling, enhances patients’ self-efficacy, with a preference for visual materials like videos and images over plain text [19,20]. Modular approaches, including basic modules for essential knowledge and additional modules for in-depth information on living with a specific disease, are also recommended [20].

The CTS Academy is based on the information provided for informed surgery consent [21], but adds further structured and comprehensive content for various stages of day surgery. Furthermore, we included aspects of mental preparation for surgery [22-26]and instructions for the postoperative period, as well as training exercises [27]. Patients can use a printable checklist for perioperative preparation and access external links, for example, the Austrian pharmacy search to locate nearby pharmacies after hospital discharge. An animated character named Karla serves as a patient role model, guiding patients through the day-surgery process and sharing important advice. To improve accessibility, the spoken text is also displayed as written text throughout the entire video.

**Figure 1.**
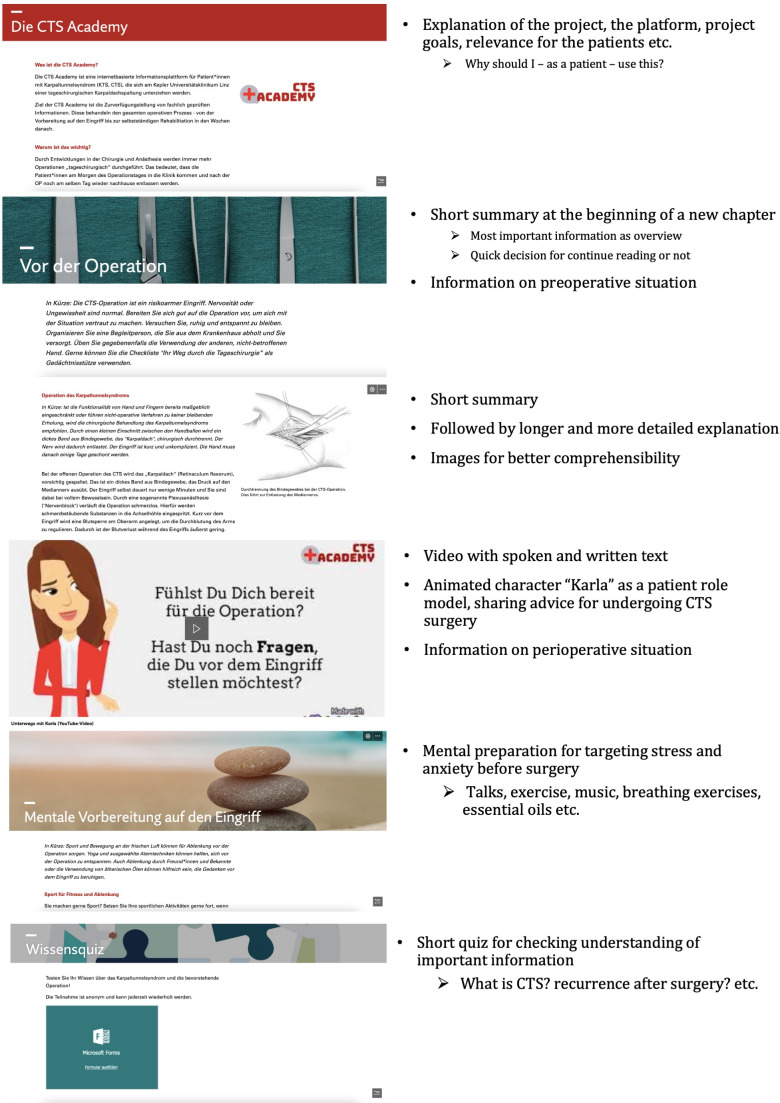
Screenshots of CTS academy including textual explanations of its content. It aims to support patients in preparing for surgery and managing postoperative recovery through self-directed online learning by offering textual information, multimedia content, self-check quizzes, and downloadable or printable checklists.

While the platform lacks structured patient assessments, it encourages self-assessment based on chapter summaries and offers knowledge checks. In Multimedia Appendix 1, we outline the content and media types presented on the CTS Academy platform. In Figure 1, screenshots of the CTS Academy platform are presented.

### Design of the Evaluation Study

Between April 2022 and April 2023, all patients presenting with CTS symptoms at the Department of Orthopedics and Traumatology and scheduled for CTR surgery after medical assessment were assessed for eligibility for inclusion in this nonrandomized, controlled, confirmatory study.

During consultations, patients received detailed information about their condition and perioperative procedures. After obtaining written informed consent for CTR, we introduced the CTS Academy study and explained the test or control groups. Allocation to the groups was based on the patient’s preference—either participation in CTS Academy or self-directed online information seeking.

Inclusion criteria were a scheduled appointment for CTR surgery, voluntariness and age of majority (18 y of age or older), physical and cognitive aptitude to participate, sufficient language skills of written and spoken German, and access to web-connected devices.

Furthermore, patients needed sufficient skills for internet use and an active pursuit of online health information.

Patients in the test group were granted unlimited access to the CTS Academy (from informed consent to surgery until 3 w postoperatively), while the control group conducted independent online searches. Throughout the self-study period of both groups, reminders were sent via SMS text messages or email to encourage information access.

Participants completed an online questionnaire after inclusion for the study, and 3 weeks postoperatively (follow-up appointment). As the CTS Academy study was conducted with German-speaking patients, all questionnaires were used in validated German versions.

The primary outcome was assessed using the Health Education Literacy of Patients with chronic musculoskeletal diseases (HELP) questionnaire by Farin, Ullrich, and Nagl [9]. It comprises 3 subtopics, that are comprehension of medical information (COMPR), applying medical information (APPLY), and communicative competence in provider interactions (COMM). Patients rated their perceived difficulty in health-related situations on a 5-point scale, with lower values indicating fewer difficulties.

Secondary outcomes included assessing the comprehensibility of content provided in the CTS Academy using the Comprehensibility Of Health Education Programmes (COHEP) questionnaire by Farin-Glattacker et al [28] and Farin, Nagl, and Ullrich [29]. This questionnaire comprises 4 subscales: transferability scale, medical information scale, amount scale, and the trainer scale, while the latter was dismissed because we did not offer lessons by a trainer [29]. Patients had to rate the amount of information provided or the perceived usefulness of information on a 6-point ranging from 1 (strongly agree) to 6 (strongly disagree).

Knowledge checks involved answering 4 statements about CTS and its treatment [30] to check their knowledge at baseline after inclusion to the study, and 3 weeks postoperatively.

Patient preferences of information seeking in the test group were assessed using the following statements on a 5-point rating scale (1=strongly disagree, 2=disagree, 3=no preference, 4=agree, and 5=strongly agree) in the follow-up questionnaire:

- I prefer the additional use of the CTS Academy (in addition to talking with a doctor) over the sole standard patient information provided in the medical informed consent conversation.
- I prefer the use of the CTS Academy over independent online information-seeking.

Usability and utility were evaluated using selected items of meCUE 2.0 [31], rated in the follow-up questionnaire on a 7-point scale ranging from 1 (strongly disagree) to 7 (strongly agree).

Clinical CTS-related parameters were examined using the validated German-language version [32] of the Boston Carpal Tunnel Questionnaire (BCTQ) [33] using symptom severity scale, functional status scale, as well as the total BCTQ score at baseline and follow-up.

Before patient testing, we conducted a pre-, pilot-, and prototype testing of the CTS Academy platform in August 2021 to examine the usability as well as to detect functionality and usability issues. This testing involved 16 voluntary participants: 11 laypersons (some with previous CTS surgery experience) and 5 experts in hand surgery, psychology, sociology, e-learning, and online didactics. Laypersons were asked to imagine themselves in the role of CTS patients undergoing surgery. Experts reviewed the platform professionally. The feedback and suggestions were discussed with the other project team members and led to updates in pre- and postoperative recommendations, as well as revisions for clarity and gender-appropriate language (shortening of the sentence length, simplifying certain terms, and standardizing gender-inclusive language). Recommendations were added regarding preoperative and postoperative smoking abstinence, as well as the ergonomic use of computer mice and keyboards after surgery. A chapter about the author of the CTS Academy was added.

### Statistical Analysis

#### Sample Size Estimation and Enrollment

We performed sample size estimation in G*Power (version 3.1.9.7) [34] using the following parameters: Wilcoxon-Mann-Whitney test (2 groups), 1-tailed hypothesis, Cohen *d* of 0.8, α error probability of 0.05, and power of 0.8 [35]. This yielded a required sample size of 21 patients per group, totaling 42 patients. Anticipating a 20% dropout rate, we aimed to recruit at least 50 patients. However, due to the COVID-19 pandemic’s impact on surgical resourced and increased dropouts, we ultimately enrolled 60 patients. Between April 2022 and April 2023, 60 patients scheduled for open CTR at our hospital participated in the CTS Academy study. Patients presenting with CTS symptoms at the hand surgery outpatient clinic underwent diagnostic evaluation, and those requiring surgery were scheduled accordingly. During preoperative consultations, they received information about the procedure, risks, and postoperative care before providing written informed consent. Eligible patients—adults with sufficient German language skills, cognitive and physical ability, regular internet use as well as willingness to seek online health information—were invited to join the study. No restrictions were placed on gender or education to ensure a representative sample. Interested patients could assign themselves to either the test group (structured web-based education) or control group (self-directed online information seeking) and received study details, including access to an online baseline questionnaire. Those in the test group were provided instructions for using the CTS Academy platform. Throughout the study, patients received reminders via email and SMS to complete study-related tasks, including the baseline and follow-up questionnaires. They were also notified before surgery and received postoperative messages encouraging engagement. Nonresponders were contacted within 2 days. Throughout the study, participants could reach the study team via phone, SMS, or email.

#### Data Exclusion and Dropouts

In our online questionnaires, all fields were mandatory except for the BCTQ functional scale, as some patients may not perform tasks specified in this BCTQ scale. Patients could review and change their answers before submitting the questionnaire. In the test group, 1% (2/144) of values were missing at baseline and at follow-up. In the control group, 6% (8/144) and 1% (2/144) were missing at baseline and follow-up, respectively. Following BCTQ guidelines, we excluded participants with more than 1 missing value, resulting in the exclusion of 1 participant from each group [36]. Since BCTQ activities are not directly comparable (eg, holding a book, doing chores, and getting dressed), missing values were not imputed.

Due to the per-protocol analysis of this study, 12 patients in each group dropped out of the study due to various reasons such as nonsubmission of questionnaires, withdrawal of consent, or canceled surgery appointments.

#### Data Preparation and Analysis

Microsoft Excel 16.72 for Mac, and IBM SPSS (version 29) were used for statistical testing.

Descriptive statistics included means and SD as well as the IQR to identify potential outliers. Normal distribution was tested using the Shapiro-Wilk-Test. The HELP questionnaire results were analyzed by summing each subscale (APPLY, COMM, and COMPR) for individual patients and transforming these sums to a 0‐100 scale. Delta values between pre- and postoperative scores (DIFF_APLLY, DIFF_COMM, and DIFF_COMPR) were calculated.

Depending on the parameters’ normal distribution, either the exact Wilcoxon test or the *t* test for dependent variables and the Mann-Whitney *U* test or the *t* test for independent variables were used for comparisons. The total HELP score (a sum of the subtypes APPLY, COMM, and COMPR) was tested at baseline, follow-up, and as difference between the former using the multivariate analysis of variances (MANOVA).

Nominal data were tested using the chi-square test or the Fisher exact test if the requirements of the former test were not met. Effect sizes were determined using Psychometrica [37].

Demographic data were examined for group comparability, focusing on age and educational level. Statistical tests were primarily 1-sided, with significance set at *P*<.05. We converted results from default 2-sided tests to 1-sided outcomes and did not perform subgroup analyses.

The COHEP questionnaire results were analyzed by summing subscales and transforming them to a 0‐100 scale. Knowledge check results were reported as the frequency of correct answers within each group. Usability and utility of the CTS Academy platform were assessed using the meCue 2.0 data template [38]. For the assessment of CTS-related symptom changes, symptom severity, functional status, and total BCTQ, of which a delta value (DIFF_BCTQ) was calculated, were compared between cohorts as well as longitudinally.

### Ethical Considerations

This study was approved and registered by the institutional review board (EK1212-2021). Written informed consent (Multimedia Appendix 2) based on the hospital’s regulative for clinical studies was obtained from every patient willing to participate in the study. To maintain data security, patients were pseudonymized by assigning each one an alphanumeric ID at the time of study enrollment. This ID was used exclusively for all surveys. A list of IDs alongside patient names was kept in the study office and was accessible to the project management team, for example, to use the reminder system (eg, reminders to complete the questionnaire). During the analysis of the results, it was not possible at any time to link the identity of the test participants to the available data. No identification of individual participants in any of the figures or additional material is possible. Patients received no compensation for study participation.

The study was reported using the CHERRIES (Checklist for Reporting Results of Internet E-Surveys) checklist (Multimedia Appendix 3) [39].

## Results

### Scoping Review

In total, 17 reports were included for full-text analysis, finding that patients searching online for CTS information risk encounter incorrect or confusing content [3], which underlines the need for reliable education programs. An overview of the results is presented in Table 1.

**Table 1.** Outcomes of the 17 references included in the scoping review on patient-centered web-based education programs focusing on the perioperative process of carpal tunnel release conducted in Epistemonikos, the Cochrane Library, PubMed, Connected Papers, and Google Scholar up to December 2022, discussing the quality of online carpal tunnel syndrome information.

Reference	Topic	Relevant results
Cook et al [40]	Complexity of online information on hand surgery	The recommended 6th-grade reading level was not met in any of the analyzed CTS^a^ material, which decreases understandability for many patients.
Goyal et al [41]	Analysis of misleading CTS information in YouTube videos	78% (47/60) of the analyzed videos contained at least 1 potentially misconception-reinforcing statement, more than 335% (21/60) contained more than 3 statements, mostly on CTS causes and treatment options.
Lutsky et al [42]	Re-evaluation of CTS information on the internet (updated study from 2000)	Significant improvement of online CTS information quality (*P*<.001), further improvement is needed.
Steimle et al [43]	Comparison of online CTS information with the Clinical Practice Guidelines of the American Academy of Orthopaedic Surgeons	Incorrect information might provoke misunderstandings and potential risks for patients.
Özbek et al [44]	Quality and reliability of CTS videos on YouTube	Significant differences in reliability and informational scores (*P*<.05) depending on the source of the video, and generally low reliability and educational quality.
Fang et al [45]	Content and design of online CTS education handouts	Handouts contained misleading information, lacked references or citations, and were complicated to understand.
Frueh et al [46]	Quality of online CTS information	Significant difference in information quality in older compared to newer CTS websites (*P*<.001). Significant difference in explaining the handling of complications (*P*=.03).
Frické et al [47]	Indicators of accuracy for online CTS information	Indicators like high Google ratings and inlinks were correlated with accuracy (*P*<.05).
Beredjiklian et al [48]	Source and content quality of online CTS information	Several websites had misleading or unconventional about CTS treatment. The mean information value score of all websites was low and differed based on authorship.
Kwak et al [49]	Quality and reliability of CTS YouTube videos	Low reliability and low educational quality of the analyzed videos.
Johnson et al [50]	Comprehensibility and applicability, cultural sensitivity, and readability of English and Spanish online CTS information material	Low comprehensibility, applicability, and readability scores, and no statistically significant differences in the overall educational quality between English and Spanish material.
Eberlin et al [51]	Readability of online CTS material	The recommended 6th grade reading level was not met in any of the analyzed CTS material, which decreases understandability for many patients.
Mert and Bozgeyik [52]	Quality and content of YouTube videos about CTS	Generally insufficient quality of content, only few videos reached good scores for global quality and accuracy.
Sproule et al [53]	Quality of online information about carpal tunnel release, Dupuytren’s fasciectomy, and trigger finger release	The majority of material provided accurate but incomplete information, and a lack of information on prognosis and complications of surgical treatment.
Ozdemir et al [54]	Quality and reliability of CTS videos on YouTube	Overall low quality of videos concerning accuracy and transparency. Lack of information on treatment risks and benefits. Significantly better scores when authored by medical centers (*P*=.02).
Park et al [55]	Accuracy of online CTS images	Only 30% (6/20) of the analyzed images had incorrect anatomical information, for example, the position of the median nerve.
Akinleye et al [56]	Readability of online hand pathology material including CTS	The recommended 6th grade reading level was not met in any of the analyzed CTS material, which decreases understandability for many patients.

a CTS: carpal tunnel syndrome.

### Patient Characteristics

**Figure 2.**
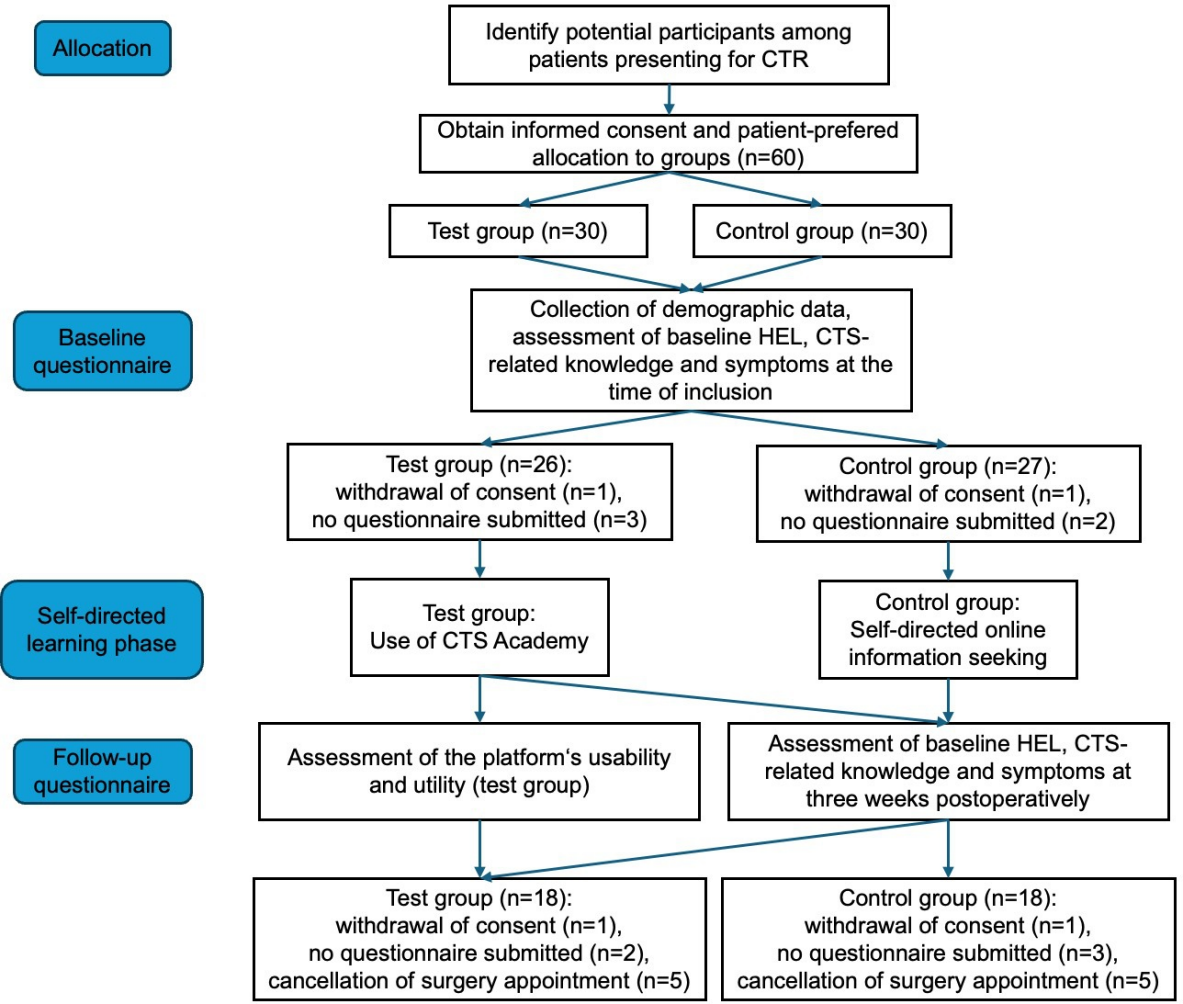
Consort flowchart of patients’ enrollment, follow-up, and analysis in the test group (CTS Academy) and control group (Self-directed online information seeking). CTR: carpal tunnel syndrome; CTS: carpal tunnel sydrome; HEL: health education literacy.

Between April 2022 and April 2023, 30 patients were enrolled in each group. Figure 2 displays a flowchart of patients including the reasons for dropout.

Ultimately, both the test and the control group comprised of 18 patients each, whose characteristics are summarized in Table 2.

The average educational phase duration (CTS Academy platform or self-directed online information seeking) was 167 (range 43-267) days, from inclusion to 176 (range 43-253) days postoperatively.

**Table 2.** Patient characteristics and demographics of the final test group (CTS Academy platform) and control group (self-directed online information seeking).

Categories and subcategories		Test group (n=18), n	Control group (n=18), n
**Sex**			
	Male	5	7
	Female	13	11
**Age group**			
	<30 years	0	0
	30‐40 years	4	3
	41‐50 years	3	3
	51‐60 years	5	2
	61‐70 years	3	6
	71‐80 years	1	4
	>80 years	2	0
**Highest education**			
	Compulsory schooling or less	2	5
	High school (without A-levels) or apprenticeship	11	9
	High school with A-levels, diploma, apprenticeship on “Master” level	4	4
	Undergraduate degree (bachelor)	1	0
	Graduate degree (master, PhD, etc)	0	0
**CTS^a^ surgery**			
	First time	14	11
	Previous CTS surgery (other hand)	4	6
	Previous CTS surgery (same hand)	0	0
	Previous CTS surgery (both hands)	0	1
**Internet use**			
	Several times a month	5	7
	Several times a week	5	3
	Daily	8	8
Average duration of CTS Academy use (days)		167 (range 43-267)	176 (range 43-253)

a CTS: carpal tunnel syndrome.

### Primary Outcome

Outcomes of the primary parameter and its subtypes are presented in Table 3 and Table 4.

**Table 3.** Outcomes (pre-post intervention analysis) of health education literacy of patients with chronic musculoskeletal diseases questionnaire (health education literacy) via subitems comprehension of medical information, applying medical information, and communicative competence in provider interactions of the final test group (CTS Academy) and control group (self-directed online information seeking) including statistical results (all *P* values are reported as 1-sided *P* values).

	APPLY^a^			COMM^b^			COMPR^c^		
	Baseline, mean (SD)	Follow-up, mean (SD)	*P* value	Baseline, mean (SD)	Follow-up, mean (SD)	*P* value	Baseline, mean (SD)	Follow-up, mean (SD)	*P* value
Test group (n=18)	28 (7.99)	24 (5.14)	.006^d^	30 (10.52)	25 (6.01)	.017^d^2	38 (16.60)	35 (9.90)	.24
Control group (n=18)	33 (14.17)	33 (14.78)	.5	35 (17.12)	30 (15.19)	.0596	50 (19.00)	46 (23.75)	.18

a APPLY: applying medical information.

b COMM: communicative competence in provider interactions.

c COMPR: comprehension of medical information.

d Statistically significant results (α<.05).

**Table 4.** Outcomes (comparison between groups [*P* values]) of health education literacy of patients with chronic musculoskeletal diseases questionnaire (health education literacy) via subitems comprehension of medical information, applying medical information, and communicative competence in provider interactions of the final test group (CTS Academy) and control group (self-directed online information seeking) including statistical results (all *P* values are reported as 1-sided *P* values).

	APPLY^a^	COMM^b^	COMPR^c^	HEL^d^
Baseline	.2	.3	.03^e^	.13
Follow-up	.04^e^	.27	.06	.05^e^
Difference	.09	.38	.37	—^f^

a APPLY: applying medical information.

b COMM: communicative competence in provider interactions.

c COMPR: comprehension of medical information.

d HEL: health education literacy.

e Statistically significant results (α<.05).

f Not applicable.

In the test group, we found a statistically significant decrease in the group means for APPLY (*P*=.006) and COMM (*P*=.02) after using the CTS Academy, compared with baseline. However, we found no statistically significant difference regarding the COMPR subparameter. An overview of statistically significant postoperative changes on item-level is provided in Multimedia Appendix 4. In the control group analysis, we found no statistically significant difference.

In the comparison between the test and control group, significantly higher APPLY values were found in the test group during the follow-up assessment (*P*=.04). For COMPR, we found the test group had significantly fewer problems with comprehension of medical information at baseline (*P*=.03).

We found a statistically significant difference between the groups on the combined dependent variables at follow-up (*P*=.05). In the subsequent post hoc univariate ANOVA for each dependent variable (between-participant effects), we found a statistically significant difference between the groups for APPLY in the follow-up examination (*P*=.007).

### Secondary Outcomes

As lower means indicate better results, we found the CTS Academy yielded good results on the transferability (mean 27, SD 9.74) and the medical information scale (mean 28, SD 10.12) and scored average results for the amount of medical information provided (mean 58, SD 24.61).

Both groups achieved high accuracy in describing CTS and the surgical procedure at baseline (63/72, 88% vs 62/72, 86% in test group and control group). At follow-up, the test group showed an average accuracy of 92%, with incorrect answers for only 1 statement. The control group yielded an average accuracy of 90% with incorrect answers for all 4 statements. No control group patient answered all statements correctly.

Regarding patients’ preference for using the CTS Academy, 10 out of 18 (56%) patients totally agreed and 6 out of 18 (33%) agreed that they preferred the additional use of the CTS Academy (in addition to the consultation of a medical doctor). Furthermore, 2 patients had no preferences*.* When asked whether they preferred the CTS Academy over independent online information-seeking, 10 out of 18 (56%) patients totally agreed and 5 out of 18 (28%) agreed, while 3 patients had no preferences.

Patients rated usability with a mean 6.22 (SD 0.82) points (4.67 to 7.00 points), and utility with a mean 6.13 (SD 0.89) points (4.00 to 7.00 points).

The CTS-related symptoms assessed via the BCTQ are displayed in Table 5.

We found no statistically relevant differences between the groups (comparison of groups at baseline: *P*=.34 [2-sided], follow-up: *P=*.47 [2-sided], and for DIFF_BCTQ: *P=*.86 [2-sided]).

**Table 5.** Carpal tunnel syndrome–related symptoms of test group and control group at baseline and follow-up, assessed with the Boston carpal tunnel questionnaire.

	Symptom severity, mean (SD)		Functional status, mean (SD)	
	Baseline	Follow-up	Baseline	Follow-up
Test group (n=18)	3.03 (0.68)	1.99 (0.71)	2.24 (0.73)	2.24 (0.87)
Control group (n=18)	3.36 (0.92)	2.19 (0.98)	2.40 (1.04)	2.48 (1.04)

## Discussion

### Principal Results

This study introduced the CTS Academy to improve patients’ HEL compared with self-directed online information seeking. Significant improvements were observed in the HELP subscales APPLY and COMM within the test group, indicating that the CTS Academy enhances patients’ ability to apply medical information and communicate with health care professionals. Patients in the study group rated both utility (usefulness) and usability (ease of use) of the CTS Academy favorably.

CTS Academy content was evaluated favorably for transferability and medical information, with average ratings for the amount of medical information provided. Development relied on existing research in clinical patient education due to a lack of comprehensive guidelines, potentially affecting perceived content quality [17]. Future improvements could involve personalized pathways based on patient knowledge levels.

Both groups showed strong understanding of CTS etiology before and after the intervention. The test group’s understanding of CTR surgery improved notably, with all participants (N=18) having all answers correct at follow-up. Misconceptions about complete symptom removal persisted in both groups, which might be due to a focus on postoperative recovery over potential complications.

Patients preferred the CTS Academy over self-directed online searches, indicating its value as a reliable information source. We conclude that integrating the CTS Academy into patient education could effectively enhance access to information and understanding of the perioperative situation, complementing discussions with medical professionals during informed consent.

High ratings for utility and usability suggest the platform is user-friendly and effective for CTR day surgery.

No significant differences were found between groups regarding perceived symptom severity or functional status, reflecting consistent surgical intervention.

The CTS Academy is the first web-based patient education program for CTS. No direct comparisons in medical literature are available, especially because no comparable programs have health education as primary outcome. Future updates to the CTS Academy will focus on restructuring content and enhancing readability and comprehensibility. The platform has the potential to evolve into an educational program with user accounts, enabling patients to follow structured, personalized learning paths. The integration of technologies such as artificial intelligence could further personalize and enhance the learning experience. Another future goal of this project is expanding the platform to include multiple languages relevant to clinical practice at our hospital.

### Comparison With Previous Work

Patient education is crucial for day surgery, as it helps prevent adverse events and rehospitalization [57]. Pekonen et al [58] conducted a systematic review on tools for measuring patient empowerment after patient education interventions. While the Health Education Impact Questionnaire is well-established [59], the HELP questionnaire specifically addresses HEL. Because the latter not just assesses knowledge acquisition but also patients’ ability to apply and understand educational interventions, it was deemed more appropriate for the CTS Academy study.

A publication by Farin-Glattacker et al [28] assessed HEL using the HELP questionnaire as an outcome measure in patients undergoing rehabilitation for chronic back pain or osteoarthritis [60]. The study group stated a high level of self-reported HEL, with the average patient experiencing slight difficulty on the APPLY and COMM scales, and slight to moderate difficulty on the COMPR scale [9]. It aligns with the findings from the CTS Academy that difficulty in understanding medical terminology appears to be a common challenge [61-64].

Regarding computer-based education on CTS, a randomized study compared knowledge levels and satisfaction scores between patients receiving the same information either using computer-based education or using face-to-face education with a medical doctor. Ultimately, the computer-based education group scored significantly higher on the knowledge questionnaire [35].

In total, 2 previous publications focused on the topic of structured web-based patient education versus self-directed online information seeking. Finkelstein and Bedra [65] conducted this comparison on hypertension knowledge in 60 patients with hypertension. The test group used a structured web-based patient education program on hypertension, while the control group searched the internet for information. The test group showed significantly higher hypertension knowledge scores compared with the baseline and compared with the control group. Semistructured interviews indicated a better learning experience in the test group, particularly for those with limited computer and internet experience. Castro et al [66] found comparable results in a similar cohort study. Thus, both concluded that web-based patient education was effective for hypertension learning, even for participants with limited internet experience [65,66].

Although health literacy is often seen as empowering, it does not automatically lead to empowerment [67]. Health education is crucial for promoting health literacy [67], with health knowledge being a key dimension [68,69] according to the conceptual model of health literacy by Sørensen et al [70]. Low health literacy levels are associated with poorer health outcomes, increased morbidity and mortality [71], highlighting the importance of patient education. Several studies have proven that multimedia education programs can improve health knowledge, such as in patients with type 2 diabetes [72]. Despite existing literature on digital health interventions for improving health literacy, only few studies specifically measure health literacy outcomes, often focusing on knowledge-related outcomes instead [73]. This distinction underscores the importance of CTS Academy’s focus on HEL.

Various publications on web-based patient education address the quality and readability of online educational materials, but few compare structured web-based patient education and self-directed online information seeking. Many examined online resources presenting orthopedic, trauma surgery, pediatrics, or cardiology content [74-76] exceed the recommended sixth-grade reading level [77], posing comprehension challenges. The quality and accuracy of online educational resources varies, with those authored by medical professionals only being slightly better than those authored by commercial organizations or laypersons [44,48,54]. Due to a lack of structured analyses of German educational online resources, quality and accuracy of online information for German-speaking CTS patients cannot be assessed. However, studies indicate that more than one-third of German-speaking orthopedic patients used the internet before their hospital appointment, and 80% (63/79) of these patients intended to gain additional knowledge of their condition and treatment options. The majority of patients performing self-directed online information seeking would recommend it to other orthopedic patients [78]. Hertling et al [79] reports that 66% (832/1262) of orthopedic patients used the internet as a source of disease-related information, and the majority found the information trustworthy and useful. However, it is unclear whether patients can assess the reliability and correctness of online information and identify incorrect information [79]. While both studies indicated widespread use and acceptance of online sources among German orthopedic patients, the quality of the information sources remains unexamined.

Comparing the comprehensibility of CTS Academy content with other programs using the COHEP questionnaire is challenging due to differing contexts. Existing studies by Farin-Glattacker et al [28], Nagl et al [60], and Finkelstein et al [65] focused on different patient populations and did not provide detailed comparative data.

Patients provided positive feedback on CTS Academy’s usability and utility, appreciating its design and functionality, facilitating effective access and utilization of educational materials. Overall, these findings suggest that the CTS Academy is a valuable tool for educating and supporting patients undergoing day surgery for CTR.

In our study, we collected postoperative clinical parameters 3 weeks after CTR. Our clinical results align with the ones published by Mack et al [80] at 10‐14 days postoperatively and Kim et al [81] and Aloi et al [82] at 14 days postoperatively, despite slight differences in patient characteristics and surgical approaches.

### Limitations

This study also involves several limitations. The study was affected by COVID-19, especially by prolonged surgery waiting times leading patients to cancel their surgery appointments. This led to a smaller sample size than intended, potentially biasing our results. Furthermore, the patient-performed allocation process may have introduced selection bias. Moreover, group sizes were relatively small, potentially affecting the generalizability of the results. We did not ask patients to monitor the duration of information-seeking or the frequency of use of the CTS Academy. In addition, the quality of information accessed by the control group was not controlled, which could have influenced their outcomes and skewed comparisons with the intervention group. However, both groups completed identical baseline questions on CTS before accessing the platform content or other online resources, ensuring a basic understanding of the scope of information. These questions required basic knowledge, independent of the depth of research or exclusive platform content. This highlights a common challenge in independent online research, that is, patients struggle to identify essential information, and available content can be difficult to interpret. While the test group benefited from a patient-centered platform design, this advantage did not extend to the fundamental information itself. To preserve the natural research and learning process, patients were not required to document their sources, as this might disrupt their learning experience. For privacy reasons and to prevent artificially inflated research activities, browser histories were not collected to avoid potential identification or bias. In future research, randomization allocation to either the test or control group should be conducted to further investigate effects of the CTS Academy platform on HEL, ensuring that the results are not affected by a selection bias. In terms of usability, common usability testing guidelines report that 5 participants already detect almost all of the possible errors in a product [83]. Therefore, the usability conclusions can be seen as valid despite of the generally decreased study’s power and generalizability.

### Conclusions

Knowledge is commonly measured as a primary outcome in patient education programs for its ease of pre and post comparisons. However, the ability to apply knowledge in daily life is crucial for effective self-management. Health literacy models emphasize both knowledge and its practical application. Hence, we recommend a comprehensive evaluation for patient education programs, beyond knowledge assessments. Our findings highlight the effectiveness of our educational intervention for patients undergoing CTR day-surgery. Despite challenges in comparing outcomes with other studies, statistical analysis favored the CTS Academy in improving overall HEL. Therefore, we rejected the null hypothesis, confirming the platform’s efficacy over self-directed online information seeking. Future research could explore the quality of German-language online educational resources by having patients document and analyze their information sources. In addition, the effectiveness of the CTS Academy following its implementation at the Department of Orthopedics and Traumatology could be assessed. Investigating patient preferences and the utility of each specific chapter could help identify the most relevant educational needs and could be analyzed on item-level of each HELP or COHEP subscale for a granulated evaluation of patients’ learning experience.

## Supplementary material

10.2196/65114 Multimedia Appendix 1 Overview of content and media types used in the CTS Academy (in order of appearance on the platform).

10.2196/65114 Multimedia Appendix 2 Informed consent.

10.2196/65114 Multimedia Appendix 3 Cherries (Checklist for Reporting Results of Internet E-Surveys) checklist.

10.2196/65114 Multimedia Appendix 4 Overview of pre- and postoperative health education literacy on item-level for the test group.

## Abbreviations

APPLY: patient’s ability to apply health-related information in an everyday life
BCTQ: Boston carpal tunnel questionnaire
CHERRIES: Checklist for Reporting Results of Internet E-Surveys
COHEP: Comprehensibility Of Health Education Programmes
COMM: patient’s ability to communicate with health care professionals
COMPR: patient’s comprehension of medical information
CTR: carpal tunnel release
CTS: carpal tunnel syndrome
HEL: health education literacy
HELP: Health Education Literacy of Patients with chronic musculoskeletal diseases
MANOVA: multivariate analysis of variances
PRISMA-ScR: Preferred Reporting Items for Systematic reviews and Meta-Analyses extension for Scoping Reviews
