# Supplementary material for: Changes in Health Education Literacy After Structured Web-Based Education Versus Self-Directed Online Information Seeking in Patients Undergoing Carpal Tunnel Release Surgery: Nonrandomized, Controlled Study
Source: JMIR Form Res. 2025 Mar 25;9:e65114. doi: 10.2196/65114 (PMC11962328; doi:10.2196/65114)
Supplement: Multimedia Appendix 1 [file formative-v9-e65114-s001.docx]

Multimedia Appendix 1: Overview of content and media types used in the CTS Academy (in order of appearance on the platform).


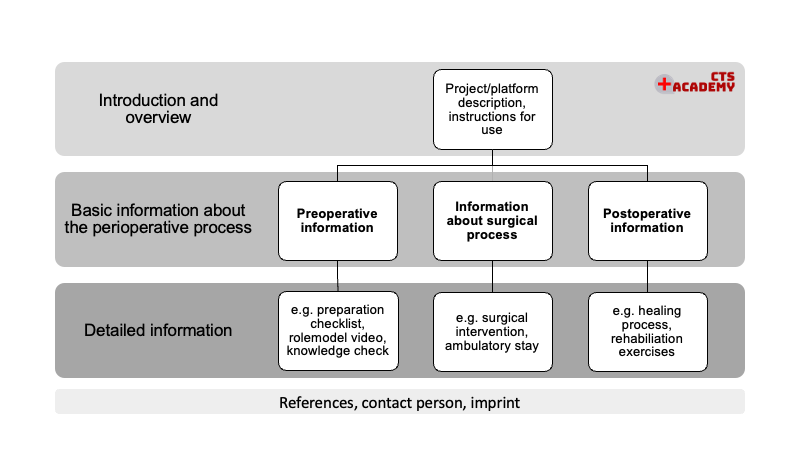


|  | Topics presented | Types of media used |
| --- | --- | --- |
| Introduction |  |  |
|  | Information about the CTS Academy platform and the research project, information about the presented content | Written structured text |
|  | *What is the CTS Academy?* |  |
|  | *Why is this important?* |  |
|  | *Why not simply use Google?* |  |
| Handling of the platform |  |  |
|  | Guidance on the handling of the CTS Academy platform, structure of chapters | Written structured text, images |
|  | *How do I use the CTS Academy?* |  |
| Education |  |  |
|  | *The carpal tunnel syndrome*: information about the medical condition (prevalence, pathogenesis, anatomy, conventional treatment) and the surgical intervention, further information about anesthesia and link to the anesthesia department at KUK | Short textual summary, written structured text, images, external web links |
|  | *The carpal tunnel syndrome* |  |
|  | *CTS surgery* |  |
|  | *Further information* |  |
| Guidance |  |  |
|  | *Before surgery*: Watch an animated video featuring patient role model Karla for insights on the hospital's day surgery process. | Short textual summary, written structured text, embedded video, printable checklist, external links |
|  | *Postoperative instructions, accompanying person for the initial 24 hours, physical preparation (smoking, adapting to using the opposite hand for eating, maintaining personal hygiene, and using walking aids), guidance for home preparation, including meal prep* |  |
|  | *Advice on critically assessing online information by checking sources* |  |
|  | *Mental preparation for surgery:* tips for getting mentally prepared and relaxed before undergoing surgery, e.g. sports, yoga and breathing techniques, finding distraction in walks and talks with close friends and family, lavender essential oils for relaxation | Short textual summary, written structured text, images, external links |
| Education |  |  |
|  | *Short quiz*: online quiz for self-assessment of patients’ understanding of the educational content | Instructional text, embedded online quiz |
| Guidance |  |  |
|  | *After surgery*: information on the postoperative situation and discharge from hospital, information on pain and the use of pain medication, drawing attention to postoperative complications such as excessive pain, bleeding or numbness and when to consult a healthcare professional, instructions for scar treatment | Short textual summary, written structured text, external links |
|  | *You have made it through surgery!* |  |
|  | *Pain* |  |
|  | *Take care: pain, bleedings etc.* |  |
|  | *Scar care* |  |
| Recovery |  |  |
|  | *Training exercises for recovery*: information on when to start with recovery exercises and tips for preventing the recurrence of CTS, e.g. recommended hand position for computer mouse and keyboard use | Short textual summary, written and graphical instructions |
|  | *Recovery*: information on the recovery process, indicating that physical recovery can vary from person to person, outline of the time frame and activities after surgery, e.g. removal of stitches, return to work, outpatient checks | Written text, images |
| Information |  |  |
|  | About the author | Written text, images |
|  | Sources of information and images used in the CTS Academy | Written text, external links |
|  | Imprint | Written text, external links |
