# Supplementary material for: Changes in Health Education Literacy After Structured Web-Based Education Versus Self-Directed Online Information Seeking in Patients Undergoing Carpal Tunnel Release Surgery: Nonrandomized, Controlled Study
Source: JMIR Form Res. 2025 Mar 25;9:e65114. doi: 10.2196/65114 (PMC11962328; doi:10.2196/65114)
Supplement: Multimedia Appendix 2 [file formative-v9-e65114-s002.pdf]

PatientInneninformation[1] und Einwilligungserklärung zur Teilnahme an der klinischen Studie

CTS Academy - Implementierung und Evaluierung einer Online-Plattform zur perioperativen Patientenschulung in der Tageschirurgie: Veränderungen in der spezifischen Gesundheitskompetenz von PatientInnen mit Karpaltunnelsyndrom-Operation am Kepler Universitätsklinikum Linz

Sehr geehrte Teilnehmerin, sehr geehrter Teilnehmer!

Wir laden Sie ein an der oben genannten klinischen Studie teilzunehmen. Die Aufklärung darüber erfolgt in einem ausführlichen ärztlichen Gespräch.

Ihre Teilnahme an dieser klinischen Studie erfolgt freiwillig. Sie können jederzeit ohne Angabe von Gründen aus der Studie ausscheiden. Die Ablehnung der Teilnahme oder ein vorzeitiges Ausscheiden aus dieser Studie hat keine nachteiligen Folgen für Ihre medizinische Betreuung.

Klinische Studien sind notwendig, um verlässliche neue medizinische Forschungsergebnisse zu gewinnen. Unverzichtbare Voraussetzung für die Durchführung einer klinischen Studie ist jedoch, dass Sie Ihr Einverständnis zur Teilnahme an dieser klinischen Studie schriftlich erklären. Bitte lesen Sie den folgenden Text als Ergänzung zum Informationsgespräch mit Ihrem Arzt sorgfältig durch und zögern Sie nicht Fragen zu stellen.

Bitte unterschreiben Sie die Einwilligungserklärung nur

- wenn Sie Art und Ablauf der klinischen Studie vollständig verstanden haben,
- wenn Sie bereit sind, der Teilnahme zuzustimmen und
- wenn Sie sich über Ihre Rechte als Teilnehmer an dieser klinischen Studie im Klaren sind.

Zu dieser klinischen Studie, sowie zur Patienteninformation und Einwilligungserklärung wurde von der zuständigen Ethikkommission eine befürwortende Stellungnahme abgegeben.

1. Was ist der Zweck der klinischen Studie?

Der Zweck dieser klinischen Studie ist die Einführung und Bewertung einer Online-Informationsplattform („CTS Academy“) auf der Webseite des Kepler Universitätsklinikums Linz. Diese Plattform dient zur Schulung von Patienten, die sich am Kepler Universitätsklinikum Linz einer Karpaldachspaltung unterziehen, um sie

[1] Wegen der besseren Lesbarkeit wird im weiteren Text zum Teil auf die gleichzeitige Verwendung weiblicher und männlicher Personenbegriffe verzichtet. Gemeint und angesprochen sind – sofern zutreffend – immer beide Geschlechter.

umfassend auf den Eingriff sowie die Situation davor und danach vorzubereiten. Die Studie untersucht primär Veränderungen in der spezifischen Gesundheitskompetenz (“health education literacy“) durch die Teilnahme an diesem webbasierten, strukturierten Schulungsprogramm. Dabei soll herausgefunden werden, ob Patientinnen, die sich mit der CTS Academy auf ihren Eingriff vorbereiten, besser über ihre Operation Bescheid wissen und medizinische Informationen besser anwenden können als jene, die sich ohne Schulungsprogramm informieren. Weiters werden auch die Verständlichkeit der Schulungsinhalte, Patientenpräferenzen, Bedienbarkeit und Veränderungen Ihrer CTS-Symptomatik untersucht.

2. Wie läuft die klinische Studie ab?

Diese klinische Studie wird an unserer Klinik durchgeführt, und es werden insgesamt ungefähr 50 Personen daran teilnehmen.

Ihre Teilnahme an dieser klinischen Studie wird voraussichtlich bis zur Operation sowie 3 Wochen darüberhinausgehend dauern.

<u>Folgende Maßnahmen werden ausschließlich aus Studiengründen durchgeführt:</u>

Während dieser klinischen Studie werden Sie nach Studieneinschluss sowie drei Wochen nach der Operation gebeten, einen umfassenden Fragebogen auszufüllen. Das können Sie bequem von Zuhause aus erledigen. Sie erhalten den Fragebogen per E-Mail zugeschickt und müssen keine studienbezogenen Besuche im Klinikum einplanen. Nach Studieneinschluss erhalten Sie Zugang zur „CTS Academy“, einer Online-Informationsplattform zur Vorbereitung auf die Karpaltunnelsyndrom-Operation und die selbstständige Versorgung danach. Sie können sich zeit- und ortsunabhängig mit dem Informationsprogramm auseinandersetzen und die Inhalte in selbstgewählter Intensität und Geschwindigkeit ansehen. Auch können Sie jederzeit Informationen nachlesen und sich zu aktuellen Anlässen im fortschreitenden Behandlungsverlauf informieren. Für den Erfolg der Studie ist es wichtig, dass Sie sich aktiv mit der Informationsplattform auseinandersetzen. Sie sollten sich zumindest einmal mit allen Inhalten zur Vor- und Nachbereitung beschäftigt haben, um die Fragestellungen in den Fragebogenerhebungen wahrheitsgemäß beantworten zu können.

Die Teilnahme am zur Verfügung gestellten Informationsprogramm (CTS Academy) sowie an den Fragebogenerhebungen ist von entscheidender Bedeutung für den Erfolg dieser klinischen Studie.

3. Worin liegt der Nutzen einer Teilnahme an der Klinischen Studie?

Es ist nicht zu erwarten, dass Sie aus Ihrer Teilnahme an dieser klinischen Studie gesundheitlichen Nutzen ziehen werden.

Wir bieten Ihnen jedoch die Möglichkeit der Nutzung eines strukturierten Schulungsangebots mit validierten und für die Patienten verständlich aufbereiteten Informationen für die geplante CTS-Operation. Sie können sich online in selbstgewählter Umgebung und Geschwindigkeit gezielt über den gesamten Operationsprozess von der

Vorbereitung bis zur selbstständigen Versorgung und Rehabilitation danach informieren. Ihr Vorteil ist dabei, dass Sie nicht den bekannten Risiken von Online-Informationen (fehlende Qualitätskontrolle, ungeeignetes Sprachniveau, fehlende Objektivität etc.) ausgesetzt sind. Die in der CTS Academy zur Verfügung gestellten Informationen wurden in Zusammenschau mit Experten verschiedener Disziplinen erarbeitet und zusammengestellt. Sie vertiefen bzw. übersteigen den Informationsgehalt der ärztlichen Aufklärungsgespräche zur OP-Einwilligung. Durch Ihre vielschichtige Beurteilung der Informationsplattform kann diese nach Studienabschluss gegebenenfalls überarbeitet und in verbesserter Weise zukünftigen Patienten zur Verfügung gestellt werden. Sie haben somit den Vorteil, ein exklusives Informationsangebot zu nutzen, zu testen und mit Ihrer Rückmeldung essentielles Feedback zur weiteren Verbesserung beizutragen.

4. Gibt es Risiken, Beschwerden und Begleiterscheinungen?

Im Rahmen dieser klinischen Studie werden keine besonderen medizinischen Maßnahmen (Blutabnahmen etc.) durchgeführt. Es ist grundsätzlich nicht mit dem Auftreten von Beschwerden oder Begleiterscheinungen zu rechnen. Die Auseinandersetzung mit den Schulungsinhalten sollte grundsätzlich ohne weitere Kontaktaufnahme mit den Behandlern möglich sein. Grundsätzlich wird die Kontaktaufnahme aber ausdrücklich empfohlen, wenn grobe Verständnisprobleme oder Fragen auftreten, die eine leitliniengemäße Durchführung der Behandlung eventuell behindern könnten. Sollten abklärungswürdige Anliegen auftreten, so ist es als positiv zu erachten, dass diese identifiziert wurden und besprochen werden. Für allfällige Fragen können Sie sich jederzeit an die angegebenen Kontaktpersonen wenden.

5. Zusätzliche Einnahme von Arzneimitteln?

Im Zusammenhang mit Ihrer Teilnahme an der klinischen Studie ist keine zusätzliche Einnahme anderer Arzneimittel als Ihrer eventuell bestehenden Medikation vorgesehen.

6. Hat die Teilnahme an der klinischen Studie sonstige Auswirkungen auf die Lebensführung und welche Verpflichtungen ergeben sich daraus?

Die Teilnahme an der klinischen Studie hat keine weiteren Auswirkungen auf Ihre Lebensführung. Es ergeben sich keine weiteren Verpflichtungen als die genannte Teilnahme am Informationsprogramm sowie an den Fragebogenerhebungen. Für den Erfolg der Studie ist es wichtig, dass Sie sich aktiv mit der Informationsplattform auseinandersetzen. Sie sollten sich zumindest einmal mit allen Inhalten zur Vor- und Nachbereitung beschäftigt haben, um die Fragestellungen in den Fragebogenerhebungen wahrheitsgemäß beantworten zu können.

7. Was ist zu tun beim Auftreten von Symptomen, Begleiterscheinungen und/oder Verletzungen?

Im Zusammenhang mit der klinischen Studie ist nicht mit dem Auftreten von Symptomen, Begleiterscheinungen oder Verletzungen zu rechnen. Sollten im Verlauf der Studie

krankheitsbezogene Verschlechterungen auftreten, kontaktieren Sie bitte Ihre behandelnden Ärzte.

9. Wann wird die klinische Studie vorzeitig beendet?

Sie können jederzeit auch ohne Angabe von Gründen Ihre Teilnahmebereitschaft widerrufen und aus der klinischen Studie ausscheiden ohne, dass Ihnen dadurch irgendwelche Nachteile für Ihre weitere medizinische Betreuung entstehen.

Ihr Studienarzt wird Sie über alle neuen Erkenntnisse, die in Bezug auf diese klinische Studie bekannt werden, und für Sie wesentlich werden könnten, umgehend informieren. Auf dieser Basis können Sie dann Ihre Entscheidung zur **weiteren** Teilnahme an dieser klinischen Studie neu überdenken.

Es ist aber auch möglich, dass Ihr Studienarzt entscheidet, Ihre Teilnahme an der klinischen Studie vorzeitig zu beenden, ohne vorher Ihr Einverständnis einzuholen. Die Gründe hierfür können sein:

a) Sie können den Erfordernissen der klinischen Studie nicht entsprechen;
b) Ihr Studienarzt hat den Eindruck, dass eine weitere Teilnahme an der klinischen Studie nicht in Ihrem Interesse ist.

10. Datenschutz

Im Rahmen dieser klinischen Studie werden Daten über Sie erhoben und verarbeitet. Mit der Einverständniserklärung erheben wir neben Ihrem Namen und Ihrem Geburtsdatum auch Ihre Telefonnummer und Ihre E-Mail-Adresse (für eine Kontaktaufnahme während der Studie und die Zusendung der Fragebögen) sowie das Datum Ihres geplanten Operationstermins (um 3 Wochen postoperativ den Abschlussfragebogen zuzusenden).

Es ist grundsätzlich zu unterscheiden zwischen

1) jenen personenbezogenen Daten, anhand derer eine Person direkt identifizierbar ist (z.B. Name, Geburtsdatum, Adresse, Sozialversicherungsnummer, Bildaufnahmen...),
2) pseudonymisierten personenbezogenen Daten, das sind Daten, bei denen alle Informationen, die direkte Rückschlüsse auf die konkrete Person zulassen, entweder entfernt, durch einen Code (z. B. eine Zahl) ersetzt oder (z.B. im Fall von Bildaufnahmen) unkenntlich gemacht werden. Es kann jedoch trotz Einhaltung dieser Maßnahmen nicht vollkommen ausgeschlossen werden, dass es unzulässigerweise zu einer Re-Identifizierung kommt.
3) anonymisierten Daten, bei denen eine Rückführung auf die konkrete Person ausgeschlossen werden kann.

Zugang zu den Daten, anhand derer Sie direkt identifizierbar sind (siehe Punkt 1), haben der Prüfarzt und andere Mitarbeiter des Studienzentrums, die an der klinischen Studie oder Ihrer medizinischen Versorgung mitwirken. Zusätzlich können autorisierte und zur Verschwiegenheit verpflichtete Beauftragte des Sponsors (Universitätsklinik für Orthopädie und Traumatologie) sowie Beauftragte von in- und/ oder ausländischen Gesundheitsbehörden und jeweils zuständige Ethikkommissionen in diese Daten Einsicht nehmen, soweit dies für die Überprüfung der ordnungsgemäßen Durchführung der klinischen Studie notwendig bzw. vorgeschrieben ist. Sämtliche Personen, die Zugang zu diesen Daten erhalten, unterliegen im Umgang mit den Daten den jeweils geltenden nationalen Datenschutzbestimmungen und/oder der EU-Datenschutz-Grundverordnung (DSGVO).

Der Code, der eine Zuordnung der pseudonymisierten Daten zu Ihrer Person ermöglicht, wird nur an Ihrem Studienzentrum aufbewahrt.

Eine Weitergabe der Daten erfolgt nur in pseudonymisierter oder anonymisierter Form. Für etwaige Veröffentlichungen werden nur die pseudonymisierten oder anonymisierten Daten verwendet.

Im Rahmen dieser klinischen Studie ist keine Weitergabe von Daten in Länder außerhalb der EU (Drittland) vorgesehen.

Ihre Einwilligung bildet die Rechtsgrundlage für die Verarbeitung Ihrer personenbezogenen Daten. Sie können die Einwilligung zur Erhebung und Verarbeitung Ihrer Daten jederzeit ohne Begründung widerrufen. Nach Ihrem Widerruf werden keine weiteren Daten mehr über Sie erhoben. Die bis zum Widerruf erhobenen Daten können allerdings weiter im Rahmen dieser klinischen Studie verarbeitet werden.

Nach der DSGVO stehen Ihnen grundsätzlich die Rechte auf Auskunft, Berichtigung, Löschung, Einschränkung der Verarbeitung, Datenübertragbarkeit und Widerspruch zu, soweit dies die Ziele der klinischen Studie nicht unmöglich macht oder ernsthaft beeinträchtigt und soweit dem nicht andere gesetzliche Vorschriften widersprechen.

Die voraussichtliche Dauer der klinischen Studie ist bis zum Wintersemester 2022/23. Die Dauer der Speicherung Ihrer Daten über das Ende oder den Abbruch der klinischen Studie hinaus ist durch Rechtsvorschriften geregelt.

Falls Sie Fragen zum Umgang mit Ihren Daten in dieser klinischen Studie haben, wenden Sie sich zunächst an Ihren Prüfarzt. Dieser kann Ihr Anliegen ggf. an die Personen, die für den Datenschutz verantwortlich sind, weiterleiten.

Kontaktdaten der Datenschutzbeauftragten der an dieser klinischen Studie beteiligten Institution: datenschutz@ooeg.at sowie datenschutz@jku.at

Für die vorliegende Studie: Mariella Seel, BA MSc

Sie haben das Recht, bei der österreichischen Datenschutzbehörde eine Beschwerde über den Umgang mit Ihren Daten einzubringen (www.dsb.gv.at; E-Mail: dsb@dsb.gv.at).

11. Entstehen für die Teilnehmer Kosten? Gibt es einen Kostenersatz oder eine Vergütung?

Durch Ihre Teilnahme an dieser klinischen Studie entstehen für Sie keine zusätzlichen Kosten. Es ist keine Vergütung Ihrer Teilnahme vorgesehen.

12. Möglichkeit zur Diskussion weiterer Fragen

Für weitere Fragen im Zusammenhang mit dieser klinischen Studie stehen Ihnen Ihr Studienarzt und seine Mitarbeiter gern zur Verfügung. Auch Fragen, die Ihre Rechte als Patient und Teilnehmer an dieser klinischen Studie betreffen, werden Ihnen gerne beantwortet.

Name der Kontaktperson: Univ.-Prof. Dr. Tobias Gotterbarm (Prüfarzt)

Ständig erreichbar unter: +43 (0)5 7680 83 - 3198

Name der Kontaktperson: Mariella Seel, BA MSc (Doktorandin)

Ständig erreichbar unter: +43 (0)677 64506239

Name der Kontaktperson: Katarina Malesevic, MSc (Studienzentrum)

Ständig erreichbar unter: +43 (0)5 7680 83 - 73056

Oö Patientenvertretung
Bahnhofplatz 1
4021 Linz
Telefon: (+43 732) 77 20 142 15
Fax: (+43 732) 77 20 21 43 55
E-Mail: ppv.post@ooe.gv.at

14. Einwilligungserklärung

Name des Patienten:

Geb.Datum:

Telefonnummer:

E-Mail-Adresse:

Geplanter Operationstermin:

Ich erkläre mich bereit, an der klinischen Studie *„CTS Academy - Implementierung und Evaluierung einer Online-Plattform zur perioperativen Patientenschulung in der Tageschirurgie: Veränderungen in der spezifischen Gesundheitskompetenz von PatientInnen mit Karpaltunnelsyndrom-Operation am Kepler Universitätsklinikum Linz"* teilzunehmen. Ich bin darüber aufgeklärt worden, dass ich die Teilnahme ohne nachteilige Folgen, insbesondere für meine medizinische Betreuung, ablehnen kann.

Ich bin von Frau/Herrn (Dr.med.) .... ausführlich und verständlich über die klinische Studie, mögliche Belastungen und Risiken, sowie über Wesen, Bedeutung und Tragweite der klinischen Studie und die sich für mich daraus ergebenden Anforderungen aufgeklärt worden. Ich habe darüber hinaus den Text dieser Patientenaufklärung und Einwilligungserklärung, die insgesamt 8 Seiten umfasst, gelesen. Aufgetretene Fragen wurden mir vom Prüfarzt verständlich und zufriedenstellend beantwortet. Ich hatte ausreichend Zeit, mich zu entscheiden. Ich habe zurzeit keine weiteren Fragen mehr.

Ich werde den ärztlichen Anordnungen, die für die Durchführung der klinischen Studie erforderlich sind, Folge leisten, behalte mir jedoch das Recht vor, meine freiwillige Mitwirkung jederzeit zu beenden, ohne dass mir daraus Nachteile, insbesondere für meine medizinische Betreuung, entstehen.

Ich stimme ausdrücklich zu, dass meine im Rahmen dieser klinischen Studie erhobenen Daten wie im Abschnitt „Datenschutz" dieses Dokuments beschrieben verarbeitet werden.

Eine Kopie dieser Patienteninformation und Einwilligungserklärung habe ich erhalten. Das Original verbleibt beim Prüfarzt.

....

(Datum und Unterschrift des Patienten)

....
(Datum, Name und Unterschrift des verantwortlichen Prüfarztes)

(Der Patient erhält eine unterschriebene Kopie der Patienteninformation und Einwilligungserklärung, das Original verbleibt im Studienordner des Prüfarztes.)
