# Supplementary material for: Changes in Health Education Literacy After Structured Web-Based Education Versus Self-Directed Online Information Seeking in Patients Undergoing Carpal Tunnel Release Surgery: Nonrandomized, Controlled Study
Source: JMIR Form Res. 2025 Mar 25;9:e65114. doi: 10.2196/65114 (PMC11962328; doi:10.2196/65114)
Supplement: Multimedia Appendix 4 [file formative-v9-e65114-s004.docx]

Multimedia Appendix 2: Overview of pre-/postoperative HEL on item-level for the test group.

|  | **Item** | **P^1^** |
| --- | --- | --- |
|  |  |  |
|  | *How much difficulty do you have in talks with doctors, therapists or nursing staff . . .* |  |
| **Applying medical information** |  |  |
|  | … distinguishing essential from less important information | .108 |
|  | … understanding medical information given to you | .094 |
|  | … understanding what the medical information means in terms of your disease | .166 |
|  | … understanding the wealth of information conveyed | .130 |
|  | … applying the medical advice you have received at home | **.008** |
| **Communicative competence** |  |  |
|  | … communicating what you already know and don’t know about your disease to doctors, therapists and nursing staff | .**004** |
|  | … communicating your own expectations and wishes in terms of your therapy | .166 |
|  | … posing very personal questions about your disease | .289 |
|  | … approaching staff when a problem of yours has not been adequately addressed | .118 |
|  | … making it clear to doctors, therapists and nursing staff how important your questions are for you | **.021** |
|  | … talking about your questions | .103 |
|  | … addressing your own problems and issues | .108 |
| **Comprehension of medical information** |  |  |
|  | … absorbing the amount of new information | .271 |
|  | … understanding complex sentences | .166 |
|  | … also understanding difficult medical information | .308 |
|  | … understanding medical information immediately | .500 |
|  | … understanding foreign words | .413 |
|  | … understanding medical terminology | .082 |
| *^1^one-tail, t-test for dependent samples, statistically significant changes (α<.05) are presented in bold* | | |
